# Why does the NHS struggle to adopt eHealth innovations? A review of macro, meso and micro factors

**DOI:** 10.1186/s12913-019-4790-x

**Published:** 2019-12-21

**Authors:** Sheena Asthana, Ray Jones, Rod Sheaff

**Affiliations:** 1 School of Law, Criminology and Government, University of Plymouth, Plymouth, UK; 2 School of Nursing and Midwifery, University of Plymouth, Plymouth, UK

**Keywords:** eHealth, Innovation, NHS, Digital policy, Barriers to eHealth

## Abstract

**Background:**

Having a tax-funded and supposedly ‘National’ Health Service (NHS), one might assume that the UK is well-positioned to roll out eHealth innovations at scale. Yet, despite a strong policy push, the English NHS has been limited in the extent to which it has exploited the potential of eHealth.

**Main body:**

This paper considers a range of macro, meso and micro factors influencing eHealth innovation in the English NHS.

**Conclusions:**

While barriers to eHealth innovation exist at all scales, the fragmentation of the NHS is the most significant factor limiting adoption and diffusion. Rather than addressing problems of fragmentation, national policy seems to have intensified the digital divide. As the recently published NHS Long Term Plan places great emphasis on the role of digital transformation in helping health and care professionals communicate better and enabling people to access the care they need quickly and easily, the implications for the digital divide are likely to be significant for effectiveness, efficiency and equity.

## Background

Developing a healthcare delivery system that is more responsive to the future challenges of an ageing population is a priority in most higher income countries experiencing late demographic and epidemiological transition. The United Kingdom (UK) is no exception. In mid-2014, the average age in the UK exceeded 40 for the first time. By 2040, nearly one in seven people is projected to be aged over 75, a trend that is likely to be accompanied by an increase in the prevalence of chronic conditions, multi-morbidities, cognitive impairments and long-term frailty [1].

The UK Government expects technology to play an increasingly important role in providing health and care support for its ageing population. The previous health and social care Secretary of State identified the need for the full integration of the health and social care system and for the National Health Service (NHS) to become “massively more teched up” [2]. His successor has listed technology as one of his top three priorities, believing that this could help achieve improvements in the other two – workforce and the prevention of illness. Their ambitions come after a series of parliamentary and government reports (most notably the recent NHS Long Term Plan [3]) that have called for the NHS to incorporate more health and medical technologies [4–7]. Yet, 10 years after Lord Darzi wrote in a national review of the NHS that “(i)n this country, we have a proud record of invention, but we lag behind in systematic uptake even of our own inventions” [8], the idea that the UK is great at generating innovations but poor at adopting them remains received wisdom.

The aim of this paper is to consider why, given such a very strong policy push, the NHS still struggles to adopt eHealth innovations. We present macro, meso and micro factors affecting innovation in eHealth, which not only act as barriers to companies seeking to commercialise and scale up their digital health businesses in the UK. Evidence of a growing digital divide between different geographical areas raises questions about the equity implications of the failure to ‘tech up’.

### What do we mean by ‘eHealth’

As there are European Union (EU) Regulations for medical devices and in vitro diagnostic medical devices as well as clear guidelines describing how the National Institute for Health and Clinical Excellence (NICE) evaluates and recommends technologies selected for appraisal, such products have a clear route to market readiness (though not necessarily to their adoption). Against this background, this paper focuses on the less specific area of eHealth.

Since earlier definitions [9–12], more recent attempts to scope digital health innovation ecosystems illustrate the growing breadth of eHealth [13–15]. This simple term can encapsulate e-health, m-health (sometimes viewed as a subset of eHealth), medicine 2.0, telemedicine and telecare, public health surveillance, personalized medicine/patient engagement, health and medical platforms, self-tracking (the quantified self), wireless health and sensors, medical imaging, healthcare information systems, mobile connectivity, social networking, sensors and wearables, gamification, electronic health records, big data, health information technology, health analytics, and digitized health systems. We would take a broader view still and include digital devices, robotics, and active assistive living [16].

### A mixed performance

With the value of the global market in mobile health alone reaching an estimated $25 billion in 2017 [17], eHealth is big business. According to analysis by Monitor Deloitte [18], the mHealth market (for health-related apps and wearable devices) is small and fragmented in the UK, accounting for a relatively low proportion of the global market. Data on consumer use of eHealth services is mixed, people in the UK being less likely to have used health apps than those in France and Italy, but more likely than in Austria and Germany. However, this is primarily to help maintain a healthy lifestyle, Britons being less likely to use health apps to manage a health condition, store personal medical data or contact a health professional than people in the other countries surveyed, suggesting less integration with formal health services [19].

The UK was an early adopter of information and communications technology (ICT) in primary care, scoring relatively well among EU member states with respect to the use of computers in General Practice [20, 21]. However, rates of electronic prescribing are now lower than in Nordic countries. There is, moreover, significant variation between Local Pharmaceutical Committees, the percentage of e-repeat prescriptions ranging from 45 to 13% in April 2019 [22].) The area where the UK has particularly fallen behind is in digital health systems and eHealth interoperability. While hospitals *departments* may have good specialist IT, many hospitals in England still lack comprehensive electronic patient record (EPR) systems [23] and the digitalisation of community health services is even further behind. This has had consequences for the sharing of information across different providers and the coordination of care.

The UK is not alone in developing national strategies or policies for eHealth, nor in encountering problems in establishing its eHealth systems. For example, the World Health Organisation WHO) recognises that progress in adopting eHealth has not been uniform across all countries in the European Region. It also notes that success in national eHealth adoption is often influenced by a range of factors that extend beyond the obvious requirements of skills and funding for technology and suggests that intersectoral engagement of stakeholders, led by the health ministry, is a key catalyst for success [24].

Having a tax-funded and supposedly ‘National’ Health Service, one might assume that the UK is well-positioned to take a lead in rolling out eHealth innovations at scale. However, the UK health market is significantly more complex than is often appreciated outside the system. First, the devolution of the NHS has resulted in significant differences in the health systems of the home countries of the UK [25]. Thus, the National Health Services of Scotland, Wales and Northern Ireland are very different to the NHS in England. Second, the English NHS is characterised by a mixture of centralisation with respect to policy setting, regulation frameworks and information governance but considerable fragmentation with respect to the organisation and delivery of care. This makes it a complex landscape for eHealth companies seeking to enter the system and scale up innovation.

### Macro scale factors influencing the adoption of eHealth innovations in the NHS

At the macro-scale, the English institutional context is ostensibly supportive of technological innovation, not least because of an interest in its scope to improve efficiency and productivity in a system that is experiencing severe financial pressures. With reported deficits in provider trusts hitting around £1 billion and an additional £213 million over-spend in commissioning organisations [26], the English NHS is increasingly described as being in a state of ‘crisis’ [27]. The last four winters (2015–18) have certainly seen numerous hospital trusts declare ‘black alerts’ about their ability to meet patient demand, with associated delays in pre-planned operations and routine outpatient appointments. There are also worrying trends in staff recruitment, retention and morale; and performance against key indicators (e.g. waiting times in Accident and Emergency and for referrals, including for cancer) appears to have worsened [27].

The current health and social care Secretary of State believes that the transformation of technology is key to releasing staff time from unnecessary bureaucracy and ensuring that different parts of the system communicate better. His pledge to invest almost half a billion pounds ($650 m) in NHS technology [28] follows several policy schemes and investments relating to innovation. Against a supportive policy context, however, there remain important structural barriers to the adoption and diffusion of eHealth innovations (see below).

#### A supportive policy context

Since the publication of the Wachter review of health technology [29], there has been a plethora of initiatives designed to speed up the adoption of innovative new drugs, devices, diagnostics and digital products in the NHS. The government’s Accelerated Access Review [6], published in 2016, introduced a newly planned Accelerated Access Pathway to prioritise strategically important innovations, an Innovation and Technology Payment to reimburse providers for a small number of selected innovations and more funding for Academic Health Science Networks to enhance local routes to market. In the same year, the Secretary of State announced a £4 billion ($5.2bn) fund to enable the NHS to be ‘paperless’ at the point of care by 2018 (this has not happened) and to be using digital, interoperable health and social care records by 2020 (this is unlikely to happen). More recently, the Office for Life Sciences launched a £35 m ($46 m) Digital Health Technology Catalyst Fund for Small and Medium Enterprises (SMEs) working in health technology. The most digitally advanced Trusts (16 acute, 7 mental health and 3 ambulance trusts) have been awarded Global Digital Exemplar (GDE) status and given matched funding of £10 m each for their digital projects; a further £200 m ($260 m) is being invested to help trusts get new IT systems off the ground; and the government is piloting a new NHS app to allow patients to book appointments and access their general practice (GP) record.

#### Digitisation and interoperability

Despite these positive policy developments, the NHS lags behind countries such as the US, with respect to basic digitisation and interoperability [30]. One possible factor may be the role that processing insurance claims has played in creating a demand for the development of electronic health records in the United States and social health insurance systems in Western Europe. Payment systems are different in the NHS, funding being distributed to the commissioners of health services (Clinical Commissioning Groups) through a central formula, while most hospital services are paid for using national tariffs. Developing standardised electronic patient record systems has not been a requirement for NHS transactions.

General practice is one area where the delivery of evidence-based clinical interventions attracts additional financial (Quality and Outcomes Framework) resources and it may be no coincidence that general practice in England is now virtually entirely digitised (though practices use several, nationally available electronic record systems, which are not standardised and not linked to other electronic health systems). By contrast, many English health and social care providers are still operating with paper records. Based on census-level data of technology adoption by English NHS hospitals, Digital Health Intelligence suggests that all NHS hospitals will not be paperless until 2027 at the earliest [31]. Progress in digitising community health and social care records has been even slower. The Department of Health and Social Care appears to have at least recognised the problem, with recent announcements that digitally less mature areas will receive central funding. It is too early to comment on what this will mean in practice.

A lack of digitisation clearly limits the ability of providers to e.g. effectively share information between patients, professionals, care settings and organisations, as does the fact that the NHS contains a plethora of incompatible patient record systems that have developed to meet the needs of *local* services or specialties. Following the disastrous National Programme for IT [24, 32] launched in 2002 and dismantled in 2011 after an investment of £7.3bn ($9.5bn), the NHS moved away from the idea of top-down mandated IT systems and instead encouraged a ‘let many flowers bloom’ approach. Local diversity is still encouraged, but, with the introduction of mandated standards for interoperability, privacy and cyber security, a “middle-out” approach is now being pursued that combines government direction with increased local autonomy [33]. Many agree that there is more hope for a genuine bottom-up change model, freed from the fetters of heavy-handed state control [34, 35]. Yet, in the absence of a clearer national steer, many commissioners lack confidence in procuring new digital systems and technologies (see below). The ‘let many flowers bloom’ philosophy is also confusing for technology suppliers.

#### Regulation and accreditation

One of the challenges facing those responsible for the planning and commissioning of NHS services for their local area is that that they are expected to draw upon robust evidence of e.g. quality, safety and cost-effectiveness to inform commissioning decisions. Despite a proliferation of eHealth technologies, few meet these evidential requirements. For example, NHS Digital (a division of the NHS) is the lead national delivery partner for improving the use of data and digital technologies in the health and care system. Yet, its Apps Library currently showcases 74 apps, a fraction of the 50,000 medical apps that are available in the Apple App Store worldwide.

This lack of evidence probably says more about methodology and existing regulatory standards than the effectiveness of eHealth innovations themselves [36]. Large pharmaceutical companies have become adept at producing cost-effectiveness analyses for the technical appraisal and licencing of pharmaceutical products, however questionable the methods and findings may sometimes be [37, 38]. Producers of medical devices are also given clear guidance about evidential requirements for technological appraisals undertaken by the National Institute for Health and Care Excellence (NICE). By contrast, the eHealth sector is dominated by small and medium sized enterprises (SMEs) many of which do not have the capacity to address the technical, clinical or cost-effectiveness standards required by NHS Digital and NICE.

For example, NHS Digital’s Digital Assessment Questionnaire comprises 12 domains and several hundred questions which, in addition to covering more technical issues such as data protection, security and interoperability, explore issues that imply a degree of evaluation, such as effectiveness, clinical safety, usability and accessibility. NICE presides over the indicators of effectiveness questions and, in its own appraisals of e.g. Improving Access to Psychological Therapies apps, tends to expect Randomised Control Trial level evidence. Thus, in the most recent evidence standards framework for digital health technologies [39], high quality observational or quasi-experimental studies demonstrating relevant outcomes are described as the minimum practice standard for demonstrating effectiveness. High quality intervention studies (quasi-experimental or experimental design) which incorporate a comparison group are considered the best practice standard.

It is questionable whether these expectations are proportionate to the needs of SMEs. Thus, those that do achieve wider roll-out tend to do so without appropriate evaluation. In July 2018, the Lancet published an editorial, calling for a clearer assessment framework in the UK for digital health, “to differentiate efficacious digital products from commercial opportunism” [40]. Insofar as a distinction is now being made between best practice and minimum evidence standards, the challenges of using traditional methods to judge the effectiveness of eHealth interventions are being acknowledged. We await clear guidance as to whether and how this translates into advice on proportionate methods of demonstrating the usability, clinical effectiveness, impact on softer outcomes etc. of eHealth technologies.

The Lancet’s reservations may have been fuelled by the rapid adoption in 2018 of Babylon Health, a health service provider that provides remote consultations with doctors and health care professionals via text and video messaging through its mobile application. The service requires a subscription fee, something many consider an affront to the NHS’s commitment to provide access to health care free at the point of use. Babylon’s symptom-checking chatbot has also attracted criticism for apparent inaccuracies in diagnosis. So far, NHS Digital has neither approved nor much influenced invention and adoption of this app. Questions have thus been raised as to whether national regulatory and indeed procurement and reimbursement systems are sufficiently agile and proportionate to support adoption at scale [41, 42]. The UK is in a strange place with, on the one hand, tight regulation of its NHS providers and, on the other, an open private market for digital providers.

### Meso scale factors influencing the adoption of eHealth innovations in the NHS

At the meso (commissioner and provider organisation) scale, important roles are played by the organisational conditions and capabilities of adopters [43, 44]. These range enormously in England, a highly fragmented healthcare system which has more than 200 Clinical Commissioning Groups (CCGs), nearly 200 provider trusts, more than 7500 primary care practices and a structural separation between the NHS (and within it, between general medical practice and community health services) and social care. This not only creates a confusing myriad of entry points for entrepreneurs and industry [45]. It has led to significant geographical variation in digital readiness, infrastructure, competencies regarding procurement and so on.

Financial pressures are also differentially distributed in the NHS and can lead to a lack of money to support innovation. Clinical Commissioning Groups (CCGs) serving older populations in rural areas are significantly more likely to be in deficit than their metropolitan counterparts, as are provider Trusts in coastal areas and rural shires [46]. There are concerns that financially struggling organisations are more likely to value the potential of eHealth innovations in narrow cost-savings terms - which are not usually easy to evidence - and to place less value on innovations that could potentially transform the quality of care. There is also a tendency to be risk averse with respect to investing in new initiatives unless they are nationally accredited or endorsed.

National policy, particularly the decision to invest in Trusts that are already digitally advanced, has exacerbated the digital divide. Recognising that there was insufficient funding to achieve the vision of a paperless NHS, leaders of the NHS IT Strategy chose to invest in a relatively small group of digitally advanced providers with a view to demonstrating what investment in NHS IT could achieve. Areas that have shown potential to go on and develop shared health and care records for the people in their wider region have been awarded Local Health and Care Record Exemplar (LHCRE) status [47]. Future investment of £37.5 m ($50 m) in new Digital Innovation Hubs which is designed to connect regional health and care data with biomedical data is expected to complement initiatives such as LHCRE. Areas such as Greater Manchester are now galloping ahead with respect to their digital health ecosystems and, as such, are attracting further investment from both Government and industry. As a result, digital maturity would appear to be geographically concentrating rather than trickling down.

### Micro scale factors influencing the adoption of eHealth innovations in the NHS

At the micro-scale, new technologies need to fit values, priorities and routines – of staff and patients [48]. Resistance to technological innovation may be based on legitimate concerns that it will lead to ‘hidden work’ [49] or undermine the quality of patient-professional interactions. Several studies suggest that, far from being convenient, Electronic Health Records can be highly labour intensive with respect to data entry. This needs to be understood within the context of the high regulatory, bureaucratic and administrative burden of the NHS, the level of data required by the system colloquially referred to as ‘feeding the beast’. Tensions between the NHS’s Information Governance framework (e.g. around patient confidentiality) and the potential benefits of e.g. record sharing can also be difficult to negotiate. Finally, there is uncertainty as to how technological developments (e.g. genomics, digital medicine, artificial intelligence (AI) and robotics) will change the roles and functions of clinical staff, which naturally gives rise to scepticism. Greenhalgh et al. [41] suggest that acceptance by professional staff may be the single most important determinant of whether a new technology-supported service succeeds or fails at a local level.

Interpersonal connections are also critical for creating the necessary trust in innovation [50]. Yet, the health and social care system in England has been characterised as one that is rich in isolated clusters in need of connectivity [51]. At very localised levels, there are many examples of innovations being championed by local clinicians in the NHS. However, wider roll-out requires opportunities for interactions between clinicians, commissioners and other health and care professionals, technology developers and patients as well as their respective networks [44, 52]. Too often, senior NHS managers and health care professionals seem to be rather suspicious of SMEs’ motives, preferring to e.g. hold on to (subsequently undeveloped) intellectual property rather than work in partnership with businesses.

Such barriers are recognised within the NHS (indeed, ‘cultural’ barriers to innovation are more likely to be highlighted in policy documents than structural issues relating to fragmentation and underfunding). For example, the recent Topol Review [53] highlights the need to appoint “clinical informatics translators” who can support leadership through chief clinical information officers and other clinical informatics professionals, and to develop broad expertise in informatics across all healthcare professionals. Cultural barriers to inter-organisational and inter-professional working will still need to be addressed, however.

## Conclusion

In this paper we have explored the apparent paradox that the NHS, a system that is often perceived to be a single, monolithic organisation, does not respond well to central policy directives such as those calling for technology to play an increasingly important role in providing health and care. Of the macro, meso and micro factors we have presented, we propose that the fragmentation of the NHS is the most important barrier to innovation, a problem that is exacerbated by current approaches to appraisal that are not sufficiently agile for rapidly evolving technologies or proportionate for SMEs. As a result, we have a proliferation of eHealth technologies at the local level, very few of which are formally evaluated and more widely marketed. This leads to localisation in roll-out, inequalities in access and replication of investment in different local areas. Variations in the digital health systems used between and within local areas are also unhelpful, not least because of the additional resources this demands of companies (e.g. who may have to pay for several application programming interfaces (APIs) for an identical product). Rather than addressing problems of fragmentation, national policy seems to be intensifying the digital divide. Companies seeking to exploit the UK’s unrealised potential with respect to digital health will find good opportunities to engage and gain traction in Greater Manchester and London and the South East. Outside these areas, the market remains precarious.

As the NHS Long Term Plan places great emphasis on the role of digital transformation in helping health and care professionals communicate better and enabling people to access the care they need quickly and easily, the implications for the digital divide are likely to be significant for effectiveness, efficiency and equity. Cross-national studies suggest that the scope of e-Health implementation mostly depends on political as opposed to economic and health-related factors [54], we would propose the need for a very strong central steer to address the deeply embedded barriers to innovation in the NHS.

## Data Availability

Not applicable.
